# Real-Time Monitoring Method for Radioactive Substances Using Monolithic Active Pixel Sensors (MAPS)

**DOI:** 10.3390/s22103919

**Published:** 2022-05-22

**Authors:** Yongchao Han, Shoulong Xu, Youjun Huang

**Affiliations:** 1 China Institute of Atomic Energy, Beijing 102413, China; hanyongchao@ciae.ac.cn; 2 School of Resource Environment and Safety Engineering, University of South China, 28 West Changsheng Road, Hengyang 421001, China; 3 Nuclear Power Institute of China, Chengdu 610213, China; jordan_huang914@163.com

**Keywords:** radioactive material, radiation, real time monitoring, detection method

## Abstract

This study presents a real-time monitoring technique for radioactive substances that meets safety management needs. We studied the accumulation characteristics of radiation response signals of monolithic active pixel sensors (MAPSs) based on their response and discrimination ability to gamma (γ) photon or neutron radiation. The radiation status of the radioactive substances was determined by monitoring the accumulation data of radiation responses. As per the results, Am-Be and ^252^Cf radiation response signals are primarily concentrated in the range of 0–70 pixels. Response signals of ^60^Co and ^137^Cs γ-ray were concentrated in two regions; there was a peak in the region with a pixel value of less than 50, and a plateau in the region with a pixel value of more than 75. Therefore, the results are able to discriminate between spectra. Furthermore, we designed a radioactivity monitoring system that is able to examine multiple radioactive materials. Its working principle is that a change in the accumulation of radioactivity monitoring data indicates a radiation change during the last accumulation cycle. This study provides vital technical support for the long-term supervision of radioactive substances.

## 1. Introduction

Regulation of radioactive materials is crucial for national security and public safety. Terrorist acts involving radioactive materials, nuclear facilities, and nuclear leakage can cause great harm to public and environmental safety. Storage and transportation of spent fuel, radiation sources, and radioactive waste require special containers and strict monitoring. Therefore, real-time radiation monitoring of radioactive storage allows for a prompt response in case of leakage/diversion/etc. Currently, there are low-power-consumption devices available that offer long-term monitoring of radiation state on the surface of radioactive substances. Complementary metal–oxide–semiconductor (CMOS) monolithic active pixel sensors (MAPSs) offer low power consumption, radiation resistance, high reliability, and cost effectiveness; in addition, they exhibit great application potential in the field of ionizing radiation monitoring [1]. In recent years, MAPS-based ionizing radiation detection has been used extensively in interventional radiology to monitor individual doses of doctors and patients [2,3,4] and charged particle track detection [5]. In addition, applying this optical device to detect nuclear radiation has attracted considerable attention [6]. Based on the response of MAPS to X-rays, the Istituto Nazionale di Fisica Nucleare (INFN) developed an active real-time pixel dosimeter with wireless transmission technology for interventional radiology to monitor individual doses of doctors [7,8]. A remote measurement device was developed for low-energy radiation detection by combining CMOS radiation detection and ZigBee wireless transmission technology [9]. Moreover, MAPSs are sensitive to neutrons, and their response signals are dependent on neutron energy and injection volume [10,11,12].

These studies demonstrate the immense potential of using MAPSs for radiation detection. The detection method is based on collecting the ionized charges generated by ionized particles in the sensitive areas of a photodiode in MAPS pixels and converting them into response signals. These response signals, also known as radiation response events, are presented as two-dimensional Gaussian peaks in the matrix data of the pixel array. Response events generated by different particles differ in size, peak value, and shape [13]. MAPS can characterize the radiation effects of ionized particles, enabling it to be used for measuring X-ray and gamma (γ)-ray exposure rates [14], and dose rate detection of low-energy γ-rays [15]. By processing pixel values with appropriate algorithms, a linear relationship can be obtained between the response data of MAPS and the radiation field dose rate [16,17]. Moreover, the response of MAPS is linear as a function of gamma-ray energy [6]. For narrow-beam photons, MAPS can obtain relatively consistent detection results with an ionization chamber detector [18]. Furthermore, CMOS MAPSs have strong radiation resistance [19], which can ensure long-term and reliable work in the radiation environment.

In this study, based on the analysis of radiation response events in MAPS, we compared the characteristics and histogram distribution of response events to study changes in the cumulative amount of response events against time, and analyzed the data characteristics of radiation response signals in the mixed radiation field. Based on the results, we proposed a method for continuous monitoring of the radiation status of radioactive substances.

## 2. Materials and Methods

### 2.1. Sensors

The experiments were conducted using the Sony IMX 222LQJ-C MAPS (Sony Corporation, Tokyo, Japan) [20], the number of effective pixels was 2.43 M pixels. Pixels were designed in a 0.18 μm CMOS process with four transistors and a pinned photodiode. The pixel pitch was 2.8 μm, and the image size was 6.4 mm × 5.8 mm. Chips were operated at an analog voltage of 2.7 V, digital voltage of 1.2 V, and interface voltage of 1.8 V. The sensor provided 8-bit data, and was integrated on the sensor board Ambarella system on chip (A5s ARM, Ambarella, Santa Clara, USA) [21] to generate readout signals and digital signal processing. The sensors were covered with a layer of opaque plastic material to help insulate them from contamination due to surrounding visible light, the thickness of the opaque plastic material was 0.11 mm, and the size was 12.8 mm × 11.6 mm. During experiments, the control systems of aperture, shutter, gain, and white balance were placed at manual, and noise reduction and exposure compensation functions were turned off. The integration time of the sensor was fixed as 40 ms.

### 2.2. Experimental Setup

**Figure 1:**
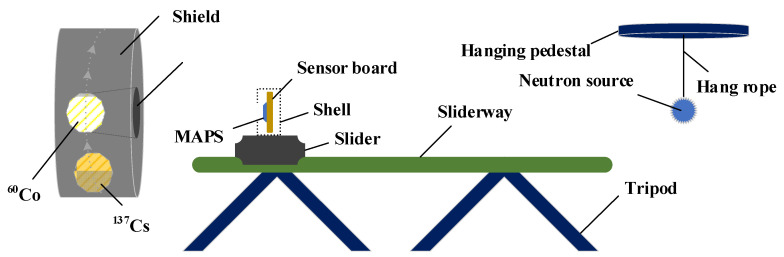
Experimental setup: the collimated ^60^Co and ^137^Cs sources; the isotropic Am-Be and ^252^Cf sources.

The experimental setup is illustrated in Figure 1. The experiment was carried out in a laboratory with 4 types of radiation sources. The sensor module was put in a container and placed in front of the collimated gamma-ray source; the diameter of the collimating hole was 8 cm. We used a facility with two collimated γ sources. We used a ^60^Co source emitting gamma photon with respective energies of 1.17 MeV and 1.33 MeV and an activity of 188 mCi, and a ^137^Cs source with photon energy of 0.662 MeV and activity of 11.3 mCi. Two encapsulated spherical isotropic sources, namely ^252^Cf and Am-Be, were used as well; the neutron emissivity of these two sources was 1 × 10^5^ neutrons/s. In particular, the alpha decay of ^241^Am in Am-Be source produces 59.5 keV γ-rays. The spontaneous fission of ^252^Cf and subsequent beta decays produce many γ-rays. These two sources were kept in a repository located below the laboratory. The radiation dose rate of γ-rays was controlled by changing the source and the distance from the source to the sensor. For detection and monitoring, we used a Thermo γ-ray detection system with a range from 0.01 μGy/h to 100 mGy/h. In the mixed radiation field experiments, two spherical isotropic sources were suspended at the same height as the sensor. The whole array of test sensors was exposed to radiation in experiments. The experimental scheme is presented in Table 1. Six groups of experiments were carried out. All experiments were conducted at the China Institute of Atomic Energy and the data were recorded online. The experimental temperature was 21°. Signal data were transmitted using 4800 LX cables, and the maximum video bitrate was 13 Mbps. For all experiments, video files were recorded at 25 fps using a computer, and the data were imported using MATLAB 2019a (MathWorks Inc., Natick, MA, USA) and then split into individual frames.

## 3. Results and Discussion

**Figure 2:**
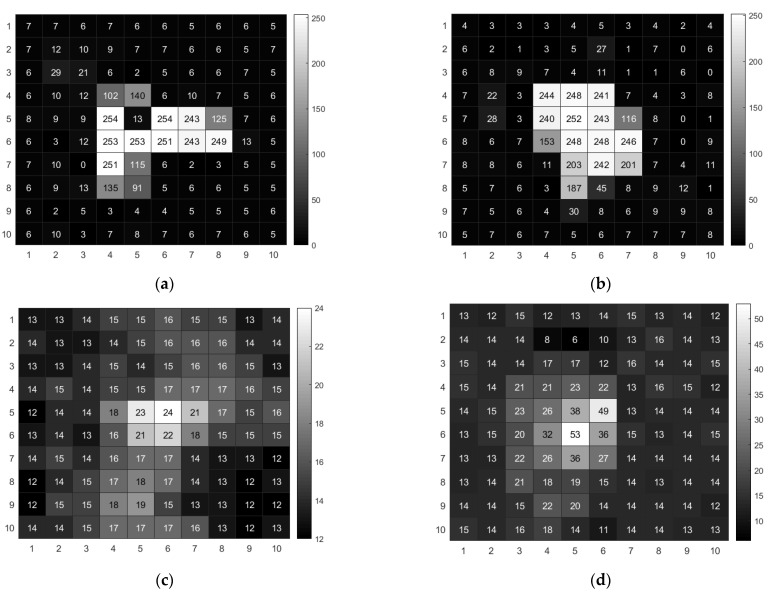
Heatmap of radiation event in a sensor frame. (**a**) ^60^Co γ source radiation Event. (**b**) ^137^Cs γ source radiation event. (**c**) Am-Be source radiation event. (**d**) ^252^Cf source radiation event.

Figure 2 displays the response events generated by different sources in a frame. As per Figure 2a,b, the response events generated by γ-rays increased pixel values dramatically within a cluster of pixels; the pixel values of non-responding pixels were close to the background. For the ^252^Cf and Am-Be radiation response event, the peak value in a cluster of pixels was much lower than that of the ^60^Co and ^137^Cs response events, and the ^252^Cf and Am-Be radiation results revealed an increase in all pixel values in the pixel array. This may be due to the strong penetration ability of γ-rays, which only produce ionized charges at the incident and surrounding pixels.

**Figure 3:**
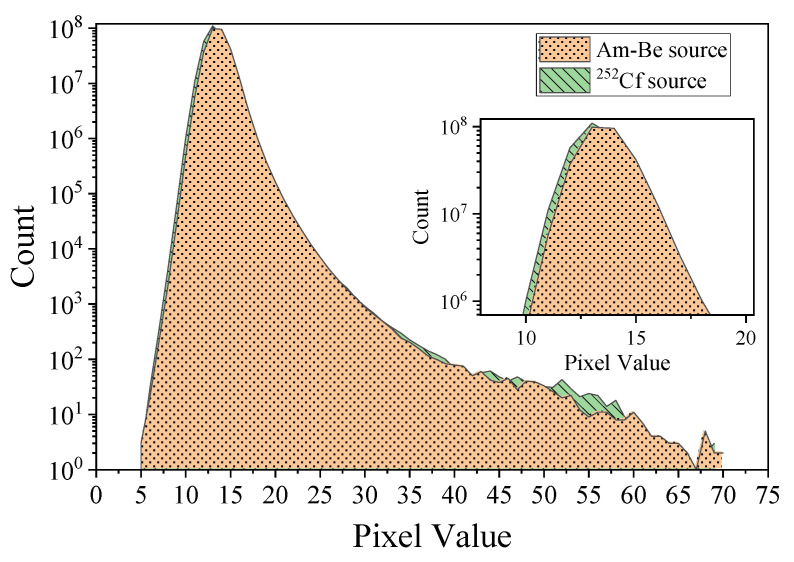
Histogram of Am-Be and ^252^Cf source radiation signals of the sensor.

Figure 3 illustrates the pixel value distribution generated in a sensor frame using the Am-Be and ^252^Cf sources. The pixel values were primarily distributed in the range of 5–70 pixels. In addition, there was only one individual distinct peak in the curves for both sources. The peak position for the ^252^Cf source irradiation curve was slightly to the right of the peak position of the Am-Be source.

**Figure 4:**
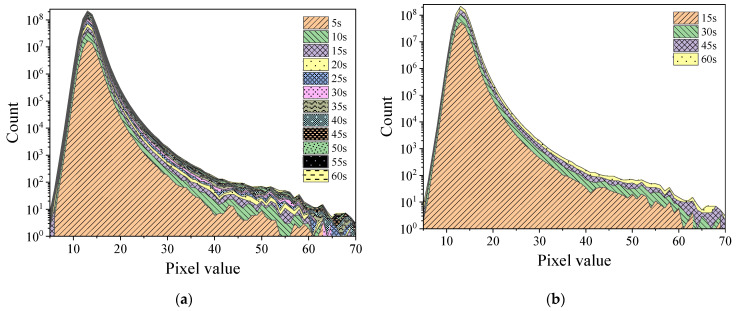
Histogram of the accumulation of Am-Be source radiation signal over time (24 frames per second). (**a**) Stacking once every 5 s. (**b**) Stacking once every 15 s.

**Figure 5:**
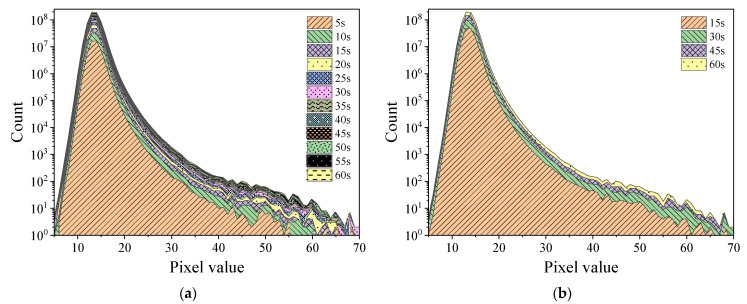
Histogram of the accumulation of ^252^Cf source radiation signal over time (24 frames per second). (**a**) Stacking once every 5 s. (**b**) Stacking once every 15 s.

Figure 4 and Figure 5, respectively, display the histograms of Am-Be and ^252^Cf source radiation signal accumulation over time. As per the figures, as frame stacking increased, a distinct accumulation of response signals was observed, indicating that the state of the radiation field can be detected continuously using the stacking of signals from frames every 5 s, 15 s, or more than 15 s. As the accumulation time extended, the value count of each pixel in the histogram increased with the superposition of frames.

**Figure 6:**
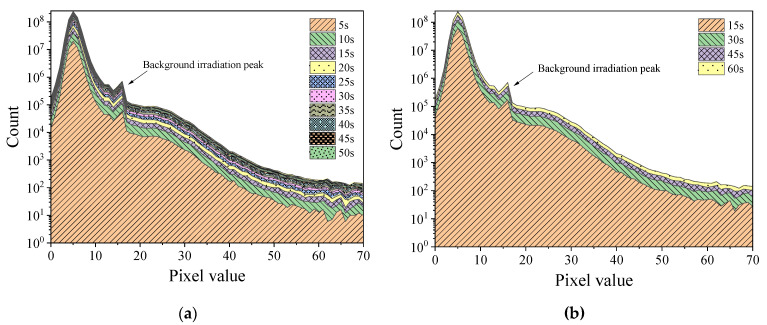
Histogram of the mixed radiation field of Am-Be source and ^60^Co source at 6.13 mGy/h (24 frames per second). (**a**) Stack once every 5 s. (**b**) Stack once every 15 s.

**Figure 7:**
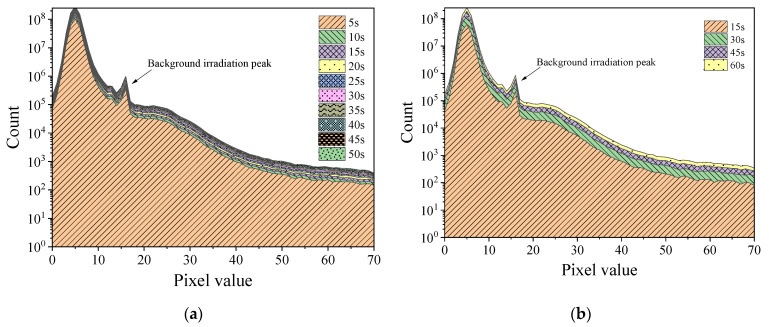
Histogram of mixed radiation field of Am-Be source and ^137^Cs source at 1.04 mGy/h (24 frames per second). (**a**) Stack once every 5 s. (**b**) Stack once every 15 s.

Figure 6 and Figure 7, respectively, display the histograms of the mixed radiation field of the Am-Be source with the ^60^Co source at 6.13 mGy/h and the ^137^Cs source at 1.04 mGy/h. Even under a mixed radiation field, the histogram exhibited the cumulative phenomenon as the time increased. In particular, a peak was observed at a pixel value of approximately 15. When compared with Figure 4 and Figure 5, this peak may have been caused by ^252^Cf or Am-Be radiation response events. Furthermore, the incremental count of each pixel value in Figure 6 was higher than that in Figure 7 over time. However, when Figure 6 and Figure 7 were compared with Figure 2a,b, respectively, there was no substantial difference in response events of ^60^Co and ^137^Cs radioactive sources, which indicates that the difference in histogram increments of Figure 6 and Figure 7 was caused by different radiation dose rates. In Figure 6, under the irradiation condition of 6.13 mGy/h, the number of response events appearing in the frame image of a specific cumulative time was more than that in Figure 7. In addition, the cumulative increment of the histogram in Figure 6 was larger.

**Figure 8:**
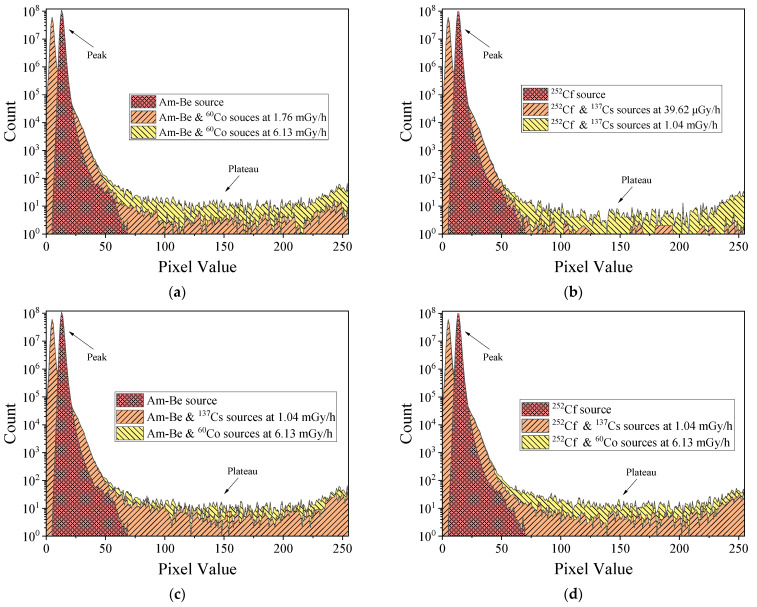
Histograms of mixed radiation signals of sensor. (**a**) Am-Be and ^60^Co sources. (**b**) ^252^Cf and ^137^Cs sources. (**c**) Am-Be and ^137^Cs or ^60^Co sources. (**d**) ^252^Cf and ^137^Cs or ^60^Co sources.

Figure 8 displays the histogram of sensor response signals under mixed radiation field conditions. The ^252^Cf and Am-Be radiation response signal was primarily concentrated in the range of 0–70 pixels. In contrast, the histogram of the double source radiation response signal had two regions, one with a peak that had a value of less than 50 pixels, and another with a plateau that had a value of more than 75 pixels. For the mixed radiation field, a part of the models in the ^252^Cf or Am-Be response region would be superimposed with the ^60^Co or ^137^Cs response signal; as a result, a range of less than 50 pixels will have two peaks, and the peak with a value of approximately 15 pixels would represent the irradiation response. The region with pixel values higher than 75 contained only ^60^Co or ^137^Cs γ-ray response signals. In the mixed radiation field, the response signal of ^252^Cf or Am-Be radiation did not interfere with that of ^60^Co or ^137^Cs γ-rays for pixel values higher than 75. In addition, any variation in histogram data was solely related to ^60^Co or ^137^Cs radiation source and radiation dose rate in that particular range. Therefore, the ^252^Cf or Am-Be source radiation status can be detected using statistical data in the range of 10–22 pixels, and ^60^Co or ^137^Cs γ radiation can be detected using statistical data in the range of pixel values higher than 75.

## 4. Application and Method

### 4.1. Detection System Setup

**Figure 9:**
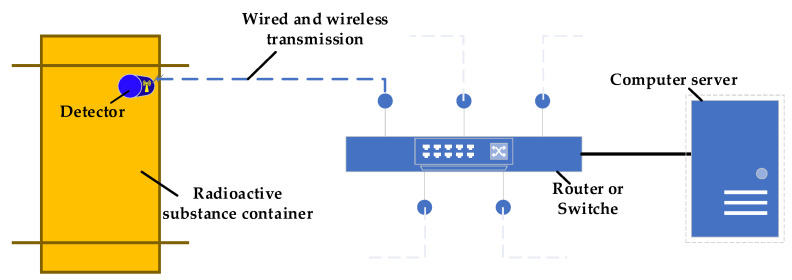
Schematic of the proposed surface radiation detection system for radioactive materials.

Figure 9 displays the schematic diagram of the proposed system designed to detect the radiation of radioactive substances. It includes a detector module, a data receiving module, and a data processing module. It can simultaneously monitor the radiation of radioactive substances. The detector module is composed of MAPSs, positioning devices, data pre-processing modules, and transmission modules, and is placed on the surface of the radioactive material. MAPS is used to collect the location information and radiation response signals of radioactive substances. The pre-processing module converts the collected frame data into histogram data, and the transmission module transmits the detection data to the network. The data receiving and processing equipment receives the detection data of each detector, and then conducts cumulative processing and issues a warning.

**Figure 10:**
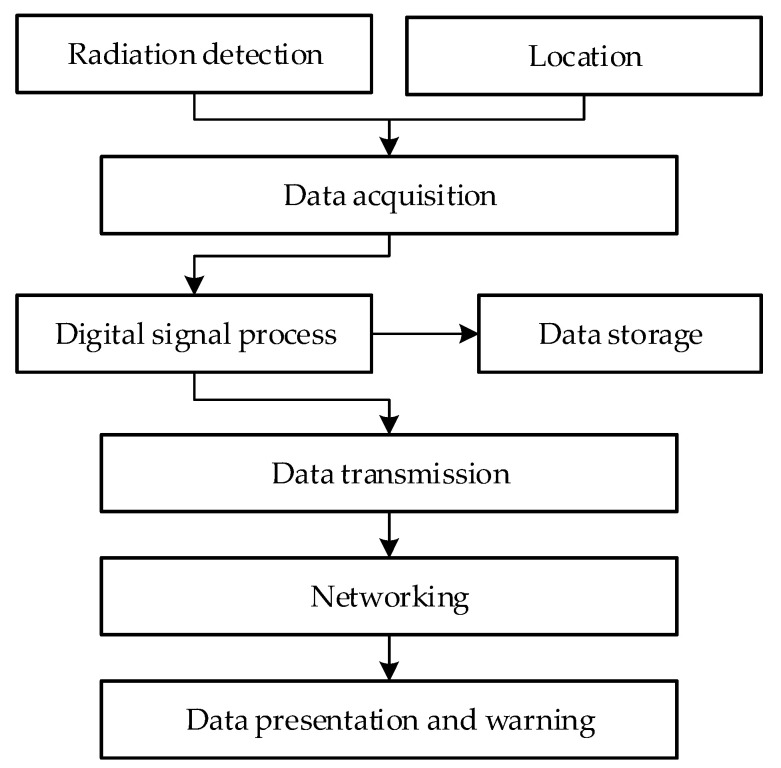
Logical block diagram of the working process of the proposed detection system.

Figure 10 displays a logical block diagram that describes the working process of the detection system. The detector collects the radiation and location information of radioactive substances in real time to perform data pre-processing. Thereafter, the original data are stored and transmitted to the network server. Lastly, the monitoring data of multiple radioactive substances are simultaneously transmitted to the computer server for cumulative processing for detection, such that abnormal data, if any, can trigger a warning.

### 4.2. Detection Method

**Figure 11:**
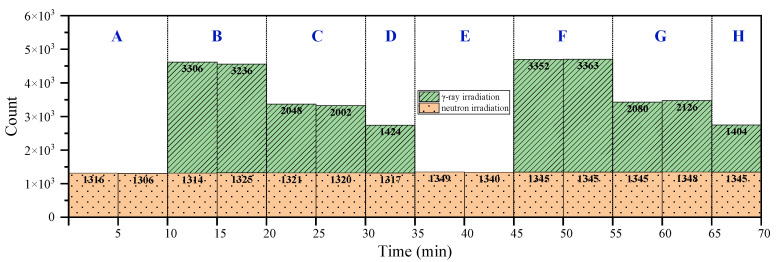
Histogram of detection data under different radiation conditions. (The time required to exchange radiation source and replacement dose rate has been excluded).

Based on the foregoing conclusions, the proposed detection system monitors the radiation of radioactive substances by counting the superposition of pixel counts in the histogram to estimate whether the radiation has changed. Figure 11 displays the histogram of radiation detected by the proposed detection system in eight different radiation fields (A–H) within 70 min; this does not include the time required to replace the radioactive source and change the radiation dose rate. Table 2 presents the corresponding radioactive sources and radiation fields of each area described in Figure 11. As per the figure, changing the radiation source or radiation dose rate caused substantial changes in the monitoring data. Although exchanging ^252^Cf or Am-Be sources caused negligible difference in the monitoring data, it can still be considered as an exchange of radiation field. The distinct difference was obtained in the monitoring results of γ-ray dose rate when a different ^252^Cf or Am-Be source radiation was used as the background radiation. MAPS was less responsive to these four types of radiation sources. Although the number of response events collected per unit time was small, monitoring every second of real-time data is not essential. Our focus was on noting changes in the cumulative data of the pixels. A substantial change in cumulative data indicated that a change in the state of the radioactive material had occurred during the last cumulative period. Therefore, the method can be used to monitor the radiation of radioactive substances.

## 5. Conclusions

Based on the response and screening ability of MAPS with respect to ionizing radiation, this study designed a method and a detection system for monitoring the radiation of radioactive substances in real time. As per the results, response events generated by ^60^Co or ^137^Cs γ-rays were considerably different from those generated by ^252^Cf or Am-Be source radiation. Due to the strong penetrating ability of γ-rays, ionized charges were generated only at the incident pixels and surrounding pixels. In the mixed radiation field, the ^252^Cf or Am-Be source radiation response signal was primarily concentrated in the range of 0–70 pixels, and superimposed with the ^60^Co or ^137^Cs γ-ray response signal. Therefore, it exhibited two peaks in the range of less than 50 pixels. The response signal of double source radiation consisted of two regions; there was a peak in the region with pixel values of less than 50 and a plateau in the region with pixel values of more than 75. Based on these results, ^252^Cf or Am-Be source radiation can be detected using statistical data within the range of 10 to 22 pixels, and the ^60^Co or ^137^Cs γ-ray radiation can be detected using statistical data within the range of more than 75 pixels. Radiation detection does not require real-time monitoring data of every second; it focuses on the exchange of cumulative data. A substantial change in cumulative data indicates a change in the state of the radioactive material during the last cumulative period. Therefore, the method proposed in this paper can realize the radiation monitoring of radioactive substances.

Meanwhile, we are very sorry for defects in the experiments in this paper. Although COVID-19 has delayed the progress of our research work, we will focus on experimental design, and further explore the unclear conclusions presented in this paper. For future research, an Am-Be source with neutron shielding material should be used to eliminate the influence of neutron radiation; this could help us to measure the radiation response from γ.

## Figures and Tables

**Table 1:** Experimental scheme.

No.	Isotropic Source	Gamma Photon Source	
		Nuclide	Dose Rate
1	Am-Be	-	-
2	^252^Cf	-	-
3	Am-Be	^60^Co	6.13 mGy/h
4	Am-Be	^60^Co	1.76 mGy/h
5	^252^Cf	^137^Cs	1.04 mGy/h
6	^252^Cf	^137^Cs	39.62 mGy/h

**Table 2:** Radiation field information.

Region No.	Isotropic Source	Gamma Photon Source	
		Nuclide	Dose Rate
A	^252^Cf	-	-
B	^252^Cf	^60^Co	6.13 mGy/h
C	^252^Cf	^60^Co	3.31 mGy/h
D	^252^Cf	^60^Co	1.032 mGy/h
E	Am-Be	^60^Co	-
F	Am-Be	^60^Co	6.13 mGy/h
G	Am-Be	^60^Co	3.31 mGy/h
H	Am-Be	^60^Co	1.032 mGy/h

## Data Availability

Data available on request due to restrictions e.g., privacy or ethical. The data presented in this study are available on request from the corresponding author. The data are not publicly available due to further research.
